# Analysis of the *Macaca mulatta *transcriptome and the sequence divergence between *Macaca *and human

**DOI:** 10.1186/gb-2005-6-7-r60

**Published:** 2005-06-30

**Authors:** Charles L Magness, P Campion Fellin, Matthew J Thomas, Marcus J Korth, Michael B Agy, Sean C Proll, Matthew Fitzgibbon, Christina A Scherer, Douglas G Miner, Michael G Katze, Shawn P Iadonato

**Affiliations:** 1 Illumigen Biosciences Inc., Suite 450, 2203 Airport Way South, Seattle, WA 98134, USA; 2 Department of Microbiology, University of Washington, Seattle, WA 98195-8070, USA; 3 Washington National Primate Research Center, University of Washington, Seattle, WA 98195-8070, USA

## Abstract

We report the initial sequencing and comparative analysis of the *Macaca mulatta *transcriptome. Cloned sequences from 11 tissues, nine animals, and three species (*M. mulatta*, *M. fascicularis*, and *M. nemestrina*) were sampled, resulting in the generation of 48,642 sequence reads. These data represent an initial sampling of the putative rhesus orthologs for 6,216 human genes. Mean nucleotide diversity within *M. mulatta *and sequence divergence among *M. fascicularis*, *M. nemestrina*, and *M. mulatta *are also reported.

## Abstract 2

Putative *Macaca mulata *orthologs for over 6,000 human genes have been sequenced from eleven tissues and three species of macaque. Macaque inter- and intraspecific nucleotide diversity is also reported.

## Background

The sequencing of genes and genomes has become a hallmark of modern molecular biology. The resulting wealth of nucleotide sequence information has fostered advances in gene discovery, the development of genome-based technologies to study gene expression and function, and a growing interest in comparative genomics. The comparison of the human genome with the genomes of closely related species has particular appeal, and there is considerable interest in identifying genomic traits that set humans apart from other primate species [1-4]. The recent growth in sequence information for the chimpanzee has fueled this interest [4]. However, beyond that generated for chimpanzee, there has been remarkably little sequence information developed for other nonhuman primate species.

The rhesus macaque (*Macaca mulatta*) is a widely used small primate model of human disease, development, and behavior. Throughout the United States, National Institutes of Health (NIH)-supported facilities house more than 25,000 nonhuman primates, including more than 15,000 rhesus macaques [5]. Each year, approximately 13,000 nonhuman primates are used for NIH-funded research, 65% of which are rhesus [5]. These animals are used principally for infectious disease, pharmacology, and neuroscience research [6]. In particular, the rhesus model is an essential tool for acquired immunodeficiency syndrome (AIDS) research and for the development of new drugs and vaccines against human immunodeficiency virus (HIV) [7,8].

We report here on our initial efforts to sequence the rhesus macaque transcriptome. The close evolutionary relationship between rhesus and human, and its widespread use as a model for human reproduction, development, and disease, make it an ideal candidate for cDNA and genome sequencing. We have constructed cDNA libraries from a selection of diverse macaque tissues and multiple animals, and we have performed single-pass sequencing on 48,642 independent clones. This sequence information has been used to generate a rhesus macaque oligonucleotide microarray and to perform comparative analyses with human.

## Results

### Sequence data collection and preliminary analysis

We prepared cloned cDNA libraries from 11 *M. mulatta *tissues derived from nine separate animals. In addition, the liver was independently sampled from one animal each of the *M. mulatta*, *M. nemestrina*, and *M. fascicularis *species. cDNA libraries were prepared by directional lambda-based cloning into *Escherichia coli *and sequenced using standard fluorescent dye-terminator chemistry. Sequencing was performed from the vector-insert junction distal to the polyadenylate sequence.

A preliminary dataset of 48,642 independent clone sequences were collected as described in Table 1. We screened and analyzed these data as described in Materials and methods. Sequence data quality was assessed using the phred algorithm [9], with a mean of 539 high-quality base-pairs per read over the entire dataset. High-quality sequence bases are defined as those with a computed phred quality value of 20 or greater (Q ≥ 20) and an expected error rate of less than 1%. Of the cloned sequences, 9,219 contain a mammalian polyadenylation consensus sequence followed by a polyadenosine tail [10]. Data meeting minimum quality criteria (*n *= 36,921) have been submitted to GenBank and contribute to all subsequent analyses. Project data and associated information are also publicly available on the project website [11].

**Table 1:** Data-collection summary

Tissue	Sequence reads
Placenta	12,033
Brain	10,511
PBMC	7,056
Spleen	6,658
Jejunum	3,840
Liver	3,744
Ileum	2,112
Lung	1,152
Ovary	672
Testis	480
Duodenum	384
	
*M. mulatta *	46,626
*M. nemestrina *	1,152
*M. fascicularis *	864
Total	48,642

We compared each macaque sequence to the mRNA RefSeq [12] component of GenBank using the MEGABLAST algorithm [13]. The most similar human sequence was identified as that reference sequence with the most significant match by bit score. In some cases, this method will identify matches between macaque and human sequences that are not orthologs, and so should be interpreted with caution. For all subsequent analyses, those macaque sequences with equally probable matches to more than one distinct human UniGene cluster have been excluded [14]. The entire dataset taken together provides a sampling of the putative macaque orthologs for 6,216 human genes (unique human LocusLink IDs), representing approximately 25% of the human gene content by recent estimate [15].

Although libraries were constructed from poly(dT)-primed cDNAs, the dataset includes a significant amount of coding sequence. Of the 6,216 unique human LocusLink IDs that were sampled in macaque, 69.3% include coding sequence (mean aligned coding length = 602 bp), whereas 30.7% include only 5' or 3' untranslated region (UTR) sequence (mean aligned UTR length = 485 bp). Of those 69.3% of genes with sampled coding sequence, the average extent of coding sequence coverage in the macaque database is 49.9% (data not shown).

### Similarity of *Macaca *transcripts with human

**Figure 1:**
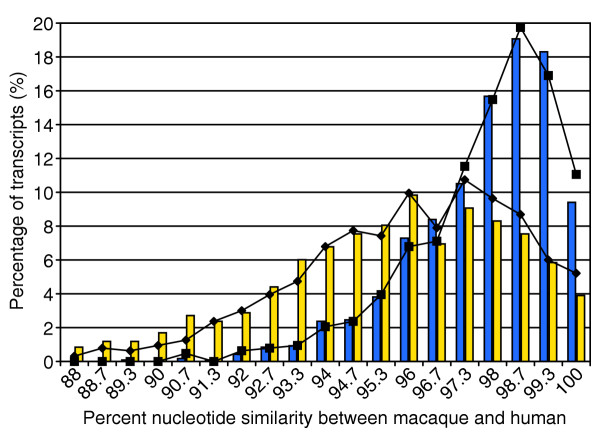
Distribution of coding and noncoding sequence similarity between macaque and human. A histogram showing the degree of nucleotide sequence similarity between macaque and human for coding (blue) and noncoding (3' UTR, yellow) transcribed sequence. Sequences (*n *= 1,180) were selected that cross a well defined stop codon and that provide concurrent sampling of 150 bp of sequence both proximal and distal to the stop. The best human match for each macaque sequence was identified using MEGABLAST. The high-quality subset of these data (composed only of contiguous stretches of phred Q ≥ 20 bp, *n *= 633) is plotted for both coding (squares) and noncoding (diamonds) sequence.

We used the initial alignment information from the above data to define a subset of sequences whose alignment with their best human match extended 150 bp in each direction around a well defined stop codon. This dataset was used to compute the distribution of sequence similarity between macaque and human as represented by the histograms in Figure 1. The use of this constrained dataset permitted a direct comparison between the distributions for coding and noncoding sequence in the vicinity of the stop codon. Data for 1,180 macaque-human alignments are included in this analysis. Sequence-similarity distributions are not normal, with a modest tail toward lower values. The average degree of similarity for coding sequence is 97.79 ± 1.78% and 95.10 ± 4.15% for the 3' UTR. This analysis excludes data where the macaque stop codon was either mutated or in a different location relative to the human reference sequence. This analysis uses the 3' UTR proximal to the stop codon as a surrogate for all untranslated sequences. However, human-chimp comparative analysis suggests that the 5' UTR may be more divergent between species than other gene regions [16]. We did not have a sufficiently sized dataset to locate and independently test conservation of the 5' UTR.

In order to determine if local regions of poor data quality contribute to biases in the computed degree of sequence similarity, we recomputed the histogram using alignments composed of only high-quality (Q ≥ 20) sequence. Constraining the dataset to include only high-quality bases (*n *= 633 sequences) did not result in significant differences in either the shape or the mean of the distributions (Figure 1).

To provide a reference dataset with which to evaluate the current results, we computed the degree of sequence similarity between human and *Pan troglodytes *(chimpanzee) using the same method as above. This analysis was performed using chimpanzee expressed sequence tag (EST) and cDNA sequences, as most currently available chimpanzee reference sequences are computationally predicted and therefore lack data from the 3' UTR. However, our chimpanzee-human analysis was hampered by the relative paucity of chimpanzee full-length cDNA and EST sequence in the public databases. There are currently only 209 full-length chimpanzee cDNA sequences and 6,930 EST sequences of varying quality in GenBank.

These data together provide a sampling of the 150 bp proximal and distal to the stop codon for only 134 human genes. On the basis of this small dataset, the degree of nucleotide identity between human and chimpanzee for coding and 3' UTR sequences is 98.3 ± 3.0% and 97.65 ± 3.2% respectively (Additional data file 1). As expected, the distribution of sequence similarity is strongly biased toward larger values, with 59.0% of sampled chimpanzee coding sequences and 46.3% of 3' UTR sequences identical to their best human match over the 150-bp window. The distribution of sequence identity between human and chimpanzee is presented in Additional data file 2.

**Figure 2:**
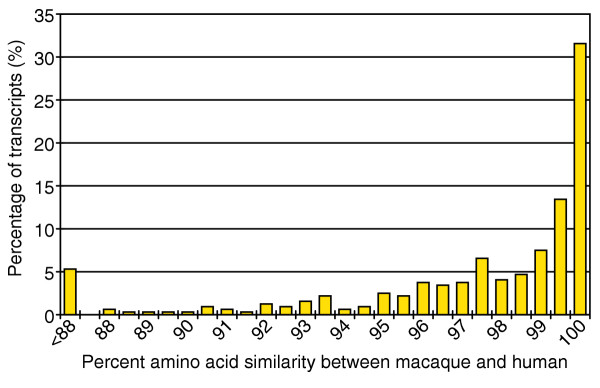
Distribution of amino-acid sequence similarity between human and macaque. Sequencing reads containing the terminal 150 amino acids of each macaque gene were compared to their best human match using MEGABLAST. Only sequences composed of contiguous high-quality bases (phred Q ≥ 20 bp, *n *= 320) throughout the terminal 150 amino acids are included. Of these sequences, 5% show less than 88% nucleotide similarity to their best-matching human homolog.

We expect that most observed nucleotide substitutions between macaque and human within coding sequence will be conservative. To evaluate the degree of similarity between human and macaque at the amino-acid level, we analyzed macaque sequences that overlapped with their best-matching human reference sequence by at least the terminal 450 bp proximal to the stop codon. Data from the terminal 450 bases were favored for this analysis in order to include more of the overall dataset and to be directly comparable to our previous nucleotide-based analysis. We also constrained the dataset to again include only high-quality bases. The distribution of amino-acid similarity was as expected, given the distribution of nucleotide similarity, with a bias toward higher values (Figure 2). The mean similarity between macaque and human protein sequences over the aligned window is 96.83 ± 4.95%. A relaxation of data quality constraints resulted in a broadening of the distribution toward lower values (data not shown).

We identified 21 high-quality macaque sequences with very weak amino-acid similarity (< 90%) to their best-matching human reference sequence (Table 2). Of these, 15 are either highly expressed in placenta or immune tissue (peripheral blood mononuclear cells (PBMCs) or spleen mononuclear lymphocytes) and/or are associated with pregnancy or the immune response. The observation of poor sequence identity for immune genes is not surprising, as increased divergence and evidence for positive selection have previously been reported for members of this group [17,18]. The most interesting example of divergence from our study is APOBEC3C, a member of the cytidine deaminase family. Rhesus macaque APOBEC3C is only approximately 85% identical to its putative human ortholog. Members of the APOBEC family are important mediators of lentivirus infection [19], and accelerated evolution has been reported for several members of this gene family [20].

**Table 2:** Macaque sequences showing weak identity with best human match

Gene	Name	RefSeq ID*	Amino-acid identity (%)^†^	Unigene ID*	LocusLink/ Gene ID*
*PSG11 *	Pregnancy specific beta-1-glycoprotein 11	NM_203287.1	68.04	Hs.502097	5680
*PSG5 *	Pregnancy specific beta-1-glycoprotein 5	NM_002781.2	73.71	Hs.534030	5673
*ANG *	Angiogenin, ribonuclease, RNase A family, 5	NM_001145.2	75.17	Hs.283749	283
*PIP *	Prolactin-induced protein	NM_002652.2	75.86	Hs.99949	5304
*GNLY *	Granulysin	NM_006433.2	76.55	Hs.105806	10578
*LAIR2 *	Leukocyte-associated Ig-like receptor 2	NM_002288.3	80.13	Hs.43803	3904
*CRYL1 *	Crystallin, lambda 1	NM_015974.1	80.31	Hs.370703	51084
*ARP10 *	ARP10 protein	NM_181773.2	82.58	Hs.440515	164668
*LOC151174 *	Hypothetical protein LOC151174	XM_371605.1	83.04	Hs.424165	151174
*GH2 *	Growth hormone 2	NM_022558.2	84.56	Hs.406754	2689
*APOBEC3C *	Apolipoprotein B mRNA editing enzyme, catalytic polypeptide-like 3C	NM_014508.2	85.26	Hs.441124	27350
*NDUFC2 *	NADH dehydrogenase (ubiquinone) 1, subcomplex unknown, 2	NM_004549.3	85.71	Hs.407860	4718
*SAA4 *	Serum amyloid A4	NM_006512.1	85.94	Hs.512677	6291
*SEPP1 *	Selenoprotein P, plasma, 1	NM_005410.1	86.07	Hs.275775	6414
*GZMB *	Granzyme B (cytotoxic T-lymphocyte-associated serine esterase 1)	NM_004131.3	86.64	Hs.1051	3002
*IFITM1 *	Interferon induced transmembrane protein 1	NM_003641.2	87.2	Hs.458414	8519
*GH1 *	Growth hormone 1	NM_000515.3	87.56	Hs.500468	2688
*TMEM14B *	Transmembrane protein 14B	NM_030969.2	87.72	Hs.273077	81853
*PRG2 *	Proteoglycan 2	NM_002728.4	88.35	Hs.512633	5553
*MRPL40 *	Mitochondrial ribosomal protein L40	NM_003776.2	88.94	Hs.431307	64976
*GKN1 *	Gastrokine 1	NM_019617.2	89.07	Hs.69319	56287

*GenBank identifiers for best matching human homolog. ^†^Amino-acid sequence identity between macaque and human.

We also identified ten placentally expressed pregnancy-related transcripts with very weak similarity to their putative human ortholog. Prominent among these are the pregnancy-specific glycoproteins (PSG5 and PSG11). For example, the best macaque match to human PSG11 shows only 68% identity and is not better matched to any other member of the human PSG family. Other placentally expressed weak orthologs include the growth mediators angiogenin (ANG) and growth hormone 1 and 2 (GH1 and GH2). Episodic accelerated evolution has previously been reported for both angiogenin and the growth hormones, although its biological and developmental implications are not well understood [21,22].

We compiled amino-acid similarity data into gene functional groupings using the 'biological process' classifications from the Gene Ontology (GO) Consortium [23] (Table 3). Data are shown for only those classes containing three or more entries. The data reveal a wide degree of variation in class-specific values of sequence similarity between human and macaque. Highly conserved classes include those involved in intracellular signaling, small GTPase-mediated signal transduction, translation initiation, and protein biosynthesis and folding. Poorly conserved biological process groups include pregnancy and immune and inflammatory response. We note that the small size of the dataset is reflected in large standard deviations for several classes of genes.

**Table 3:** Mean amino-acid identity by GO ontology

Biological process group	Mean identity (%)*	Standard deviation
Pregnancy	80.8	11.7
Cell proliferation	92.7	3
Immune response	93.9	4.1
Negative regulation of cell proliferation	94	6.2
Regulation of cell cycle	94.3	5.9
Response to oxidative stress	94.3	6.7
Inflammatory response	94.4	4.3
Transport	95	3.1
Cell-cell signaling	95.5	3.6
Apoptosis	95.6	5.3
Proteolysis and peptidolysis	96.1	4.5
Positive regulation of cell proliferation	96.2	2.4
G-protein coupled receptor protein signaling pathway	96.3	5.5
Electron transport	96.3	2.3
Development	96.4	4.1
Carbohydrate metabolism	96.7	3.2
Metabolism	96.9	2.4
Signal transduction	97	4
Cell growth and/or maintenance	97.2	3.8
Angiogenesis	97.3	2
Regulation of transcription from Pol II promoter	97.7	2.4
Mitosis	97.7	1.8
Ubiquitin cycle	97.7	3.8
Antimicrobial humoral response (sensu Vertebrata)	97.8	1.9
Ribosome biogenesis	98	1.9
Ion transport	98.1	0.6
Cell adhesion	98.3	2.9
Anti-apoptosis	98.5	1.7
Ubiquitin-dependent protein catabolism	98.7	1.3
Regulation of transcription, DNA-dependent	98.7	1.4
Protein folding	98.8	1.5
Translational initiation	99	1.7
Protein biosynthesis	99.1	1.7
Response to stress	99.4	0.5
Intracellular protein transport	99.4	0.7
Glycolysis	99.6	0.3
Nuclear mrna splicing, via spliceosome	99.6	0.3
Small gtpase mediated signal transduction	99.7	0.5
Protein transport	99.9	0.3
Intracellular signaling cascade	100	0
		

*Mean identity between group members and their best matching human homologs.

These data share similarity with recent comparative analyses between human and chimpanzee [4,24]. For example in chimpanzee, a high degree of sequence conservation and low rates of nonsynonymous substitution were found for several biological classes, including protein transport, small GTPase-mediated signal transduction, regulation of DNA-dependent transcription, intracellular signaling, and glycolysis. However, not all biological functional groups demonstrate consistent conservation among the three species. For example, the signal transduction biological class is highly conserved between chimpanzee and human, whereas its conservation between macaque and human does not significantly deviate from the mean over all classes.

### Sequence divergence within and among macaque species

Our dataset includes sequence data from nine *M. mulatta*, one *M. fascicularis*, and one *M. nemestrina*. The breadth of the dataset provides an opportunity to conduct a preliminary analysis of the polymorphism frequency within *M. mulatta *and the degree of nucleotide divergence between macaque species. We estimated the polymorphism frequency within *M. mulatta *by assembling sequencing reads from multiple animals for the same gene using phrap [9]. Polymorphisms were identified by a modified version of phred that calls two alleles at each base in the assembly and assigns each allele a quality score based on combined phred quality values (C.M., unpublished work). High-scoring polymorphisms were manually verified and are presented in Table 4 for a sample of 24 genes. This analysis includes both coding and noncoding transcribed sequences. The average nucleotide diversity (π) for this gene set in *M. mulatta *is 15.8 ± 12.5 × 10^-4 ^[25]. A large standard deviation in nucleotide diversity across genes is consistent with reports from other primate species [26-28]. The animals included in this analysis were primarily bred from wild-caught parents of Indian origin. A more comprehensive determination of nucleotide diversity will require sequence data from a greater number of genes and animals from multiple geographic locations.

**Table 4:** Estimate of *Macaca mulatta *nucleotide diversity

Gene	Comparative length	Number of animals	Nucleotide diversity (π)
*ACTB *	1,067	5	0.00110
*ACTG1 *	708	6	0.00290
*APOA1 *	746	2	0.00200
*APOA2 *	431	2	0.00350
*ATF4 *	469	4	0.00160
*B2M *	439	7	0.00000
*C15orf15 *	860	3	0.00117
*CAP1 *	667	5	0.00127
*CCNI *	547	4	0.00000
*CDC10 *	693	3	0.00190
*CTSB *	967	4	0.00078
*EEF1A1 *	865	7	0.00235
*EEF1G *	771	6	0.00000
*ENO1 *	793	5	0.00100
*FTH1 *	775	5	0.00100
*PPID *	891	3	0.00148
*RPL14 *	657	5	0.00457
*RPL15 *	631	4	0.00264
*RPL3 *	796	6	0.00339
*RPS20 *	483	4	0.00155
*SLC25A5 *	749	3	0.00088
*TPT1 *	740	7	0.00000
*TXNIP *	614	3	0.00000
*UBC *	824	6	0.00281
		Mean	0.00158
		SD	0.00125

We were also able to evaluate the degree of nucleotide sequence divergence between the three macaque species for a sample of 21 genes in this dataset (Table 5). Phred and phrap were again used to assemble overlapping sequences from multiple species and to identify species-specific variants that were then manually confirmed. Given the high degree of nucleotide similarity among the species and the small sample size, the three species did not differ beyond the measured standard deviations. However, *M. mulatta *and *M. fascicularis *appear more closely related to each other than either is to *M. nemestrina*, with an average sequence divergence between the two of 0.380 ± 0.380%. The degree of sequence divergence between *M. mulatta *and *M. nemestrina *is 0.588 ± 0.438% and 0.522 ± 0.419% between *M. fascicularis *and *M. nemestrina*. However, the dataset is not large enough for any of these pairwise differences to reach statistical significance.

**Table 5:** Interspecies substitution rates

Gene	Alignment length	Number of reads			Frequency per kilobase		
			
		*M f. *	*M.m. *	*M.n. *	*m *vs n*	*m *vs f*	*n *vs f*
*ADH1B *	819	8	5	14	0.00	0.00	2.44
*AFP *	537	1	3	1	11.17	7.45	3.72
*ALB *	2047	> 20	> 20	> 20	0.00	0.49	0.49
*AMBP *	731	1	5	4	4.10	1.37	5.47
*ANGPTL3 *	371	1	1	2	2.70	0.00	2.70
*APOA1 *	746	10	76	2	10.72	4.02	5.36
*APOA2 *	431	3	4	5	6.96	4.64	2.32
*APOC4 *	312	3	1	1	16.03	12.82	16.03
*APOE *	217	2	2	2	4.61	4.61	0.00
*APOH *	1007	4	14	14	2.98	0.00	2.98
*B2M *	587	1	90	1	0.00	11.93	11.93
*EEF1A1 *	920	7	>20	7	0.00	0.00	0.00
*FGA *	379	3	6	1	7.92	0.00	7.92
*FGB *	407	3	43	11	2.46	0.00	2.46
*FGG *	694	3	24	9	1.44	1.44	2.88
*HPR *	567	2	20	12	3.53	1.76	1.76
*RPL9 *	680	1	35	1	4.41	4.41	0.00
*SERPINC1 *	787	1	3	1	1.27	2.54	1.27
*TTR *	599	5	2	6	5.01	3.34	5.01
*UBC *	460	1	40	1	0.00	0.00	0.00
*UGT2B7 *	228	1	13	1	8.77	0.00	8.77
				Mean	5.88	3.80	5.22
				Median	3.53	1.44	2.70
				SD	4.38	3.80	4.19

*Pair wise interspecies substitution frequencies computed on a gene-by-gene basis *M.f.*, *Macaca fascicularis*; *M.m.*, *M. mulatta*; *M.n.*, *M. nemestrina*.

### Putative rhesus sequences without human orthologs

Analysis of the entire dataset revealed a small number of transcribed macaque sequences that had little or no sequence similarity to any human cDNA or genomic sequence (Table 6). We speculate that some of these macaque sequences are without orthologs in the human genome. The observation of species-specific transcribed sequences among the primates is consistent with recent comparative analysis between human and chimpanzee [4,29]. Although an absolute determination of species specificity will require a complete macaque genome sequence, we conducted preliminary computational and PCR-based analyses to test the presence or absence of these sequences in the human and other primate genomes.

**Table 6:** Macaque sequences without apparent human ortholog

Class	GenBank Accession	Ortholog by MEGABLAST*		PCR product length^†^	PCR^‡^	
				
		Human genome	Human EST		Macaque genome	Human genome
I	CX078602	Yes/93%^§^	No	98	+	-
I	CX078592	No	No	111	+	+
I	CX078596	Yes/93%^§^	No	123	+	+
I	CB552301	No	No	107	Indeterminate	Indeterminate
II	CX078598	No	No	103	+	-
II	CX078591	No	No	111	+	-
III	CB555845	No	No	127	+	Indeterminate
III	CB552531	No	No	90	+	-

*Defined as identity greater than three standard deviations below the mean. ^†^Primer sequences are available in Materials and methods. ^‡^Tested under a variety of thermal cycling conditions and annealing temperatures. ^§^Borderline identity values are displayed.

As above, we used MEGABLAST to test each macaque nucleotide sequence for one or more significant hits to the human EST or genome databases. The absence of an orthologous human sequence was defined as either no significant MEGABLAST hit in the human subset of GenBank or hits with sequence identity less than three standard deviations below the mean as measured over the entire dataset (Figure 1). Because the data were not normally distributed, the identity cutoff (approximately 92.2%) was computed using the geometric mean, which relies on a logarithmic transformation of the data. All sequences meeting this cutoff definition were also outliers based on Tukey's test [30].

We selected eight of the resulting macaque sequences for PCR-based analysis using a number of primate and human genomes (Table 6, Figure 2). The purpose of this analysis was simply to verify the presence or absence of the observed sequences in a panel of primate genomes. Selected primers had an average computed annealing temperature of 59.6 ± 0.9°C with an average amplified length of 108 ± 12 bp (Materials and methods). For each primer pair, PCR analysis was conducted at several annealing temperatures between 55 and 60°C. Genomic DNA was selected from independent *M. nemestrina *and *M. mulatta *animals in order to confirm the presence of these sequences in multiple independent genomes. Of the eight tested primer pairs, two resulted in amplification of consistent bands in both human and macaque genomic DNA, two were indeterminate in human but present in the macaques, and four, while obviously present in the macaque genomes, resulted in no consistent human-specific product under any cycling conditions.

The eight tested sequences fall generally into three categories: those with weak sequence similarity to the human genome or human-derived ESTs (class I), those with weak sequence similarity only to genes and proteins from nonhuman species (class II), and those with no significant amino-acid or nucleotide sequence similarity to any GenBank nucleic acid or protein sequence (class III).

**Figure 3:**
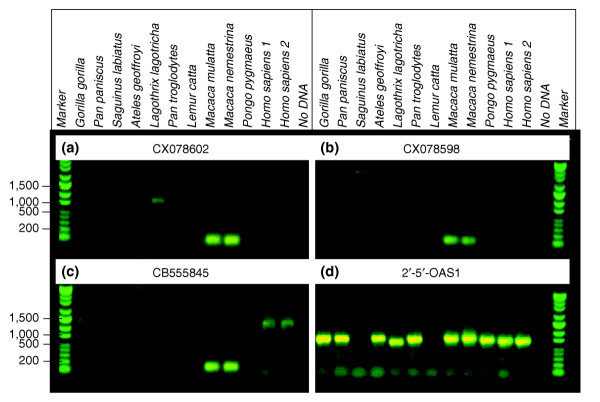
PCR analysis of putative macaque-specific sequences. PCR primers were developed from high-quality macaque cDNA sequences - **(a) **CX078602, **(b) **CX078598, and **(c) **CB555845 - and used to test for the presence or absence of the resulting amplicons in genomic DNA from 12 primate genomes, including two separate humans. Amplification conditions were the same as in Materials and methods, except that annealing was performed at 55°C. Expected product sizes are as in Table 6. **(d) **Amplification primers from exon 4 of the human oligoadenylate synthetase 1 gene (*OAS1 *) are included as a positive control, resulting in the expected 648-bp product from most primate species.

Those with weak similarity to human sequences (class I) include CX078602, a 657-bp cDNA sequence derived from macaque liver with 79-87% nucleotide sequence identity to CYP2C18 from several mammalian species. Its closest matches to human are two regions of 86-93% identity to human chromosome 10, one of which contains four cytochrome P450 2C genes. PCR-based analysis failed to amplify a consistent band from any primate species other than *M. nemestrina*, *M. mulatta*, and *Lagothrix lagotricha *(woolly monkey) (Figure 3a).

Likewise, CX078592 from brain demonstrated 88-90% nucleotide similarity to the IL15RA gene and other immune-derived transcripts, as well as to a region of human chromosome 10 containing *IL15RA*. PCR primers derived from this sequence amplified multiple specific products from macaque, human, and other primates (data not shown). Similarly, CX078596 from placenta, although having no significant match to any human EST, demonstrated significant similarity to a region of human chromosome 22. CX078596 contained a clear mammalian polyadenylation signal and poly(A) tail, and primers derived from this sequence amplified an appropriately sized product from macaque. Alignment of this sequence with human chromosome 22 revealed a 284-bp insertion in human relative to macaque, which was reflected by amplification of a proportionately larger product in two human genomic DNA samples (data not shown). Finally, although CB552301 from spleen demonstrated significant sequence identity to regions of human chromosomes 4 and 15 and multiple ESTs from UniGene cluster Hs.459311, we failed to amplify a specific product from any primate species using primers derived from this sequence (data not shown).

The second class of sequences (class II) in Table 6 had no identified human match, while demonstrating weak sequence identity to nucleic acid or protein sequences from other species. For example, CX078598, a 670-bp transcript from PBMCs, demonstrated weak amino-acid identity (67%) to the endogenous retrovirus (ERV)-BabFc^env ^envelop polyprotein, a member of the ERV-F/H family of primate retroviruses [31]. PCR with primers derived from CX078598 under a variety of thermal cycling conditions resulted in the consistent amplification of a product of expected size from only *M. mulatta *and *M. nemestrina *(Figure 2b). Similarly, CX078591 from macaque brain demonstrated weak amino-acid identity (20-45%) to ariadne homolog 2 (ARIH2/TRIAD1) from rodents and to two unnamed proteins from the puffer fish *Tetraodon nigroviridis*. Primers derived from this sequence amplified the appropriately sized product only from macaque genomic DNA (data not shown).

The last class of sequences (class III) in Table 6 demonstrated no significant similarity to any protein or nucleotide sequence in GenBank (represented by CB555845 and CB552531). Both showed evidence of a mammalian polyadenylation consensus sequence near their 3' terminus, with CB552531 additionally demonstrating a clear poly(A) tail. CB555845, a 485-bp sequence from spleen, amplified expected products from both *M. nemestrina *and *M. mulatta*. However, this clone was ultimately scored as indeterminate because of its consistently weak amplification of a discrete product from all hominids including human (Figure 2c). CB552531 amplified products of the expected size from macaque species and from *Ateles geoffroyi *and *Lemur catta*, but not from human (data not shown).

It is important to note that PCR-based analysis of divergent sequences is subject to a variety of influences and may result in different conclusions under different conditions. Furthermore, we cannot rule out the possibility that one or more of the sequences in Table 6 are alternatively spliced relative to human, pseudogenes, or genomic DNA contamination. However, each clone sequence in Table 6 demonstrated similarity to known expressed sequences or a polyadenylation consensus sequence and poly(A) tail at their 3' terminus upon complete sequencing of the clones.

### Development of a macaque-specific expression microarray resource

Genome-based technologies such as DNA microarrays are now commonplace in human biomedical research. Similarly, species-specific arrays exist for model organisms such as the mouse and rat, for which a considerable amount of genome information is available. In contrast, researchers wishing to carry out gene-expression analyses on nonhuman primate cells or tissues are currently forced to use human DNA microarrays. As part of our effort to bring genome-based technologies to researchers using nonhuman primates, we have used ESTs generated by this project to construct a rhesus macaque-specific oligonucleotide microarray.

Oligonucleotides were designed as described in Materials and methods and arrayed onto glass slides by Agilent Technologies. Briefly, macaque cDNA sequences were assembled into 9,344 distinct clusters using The Institute for Genome Research (TIGR) clustering tools [32]. From these, 7,973 macaque-specific oligonucleotide probes were identified for inclusion on the array. These probes represent the putative macaque equivalent of 3,519 unique human UniGene clusters [14] and 3,045 unique human RefSeqs [12]. To quality control the microarray, we measured tissue-specific differences in gene expression as a means of evaluating whether the oligonucleotides were successfully binding target sequences. For these experiments, we hybridized the microarray with probes derived from RNA isolated from various rhesus macaque tissues. Probes were paired in different combinations and two dye-flipped technical replicates were performed for each pair of samples. Of the 7,973 rhesus macaque oligonucleotides present on the microarray, 6,215 showed differential expression (equal or greater than twofold; *P *≤ 0.01) in at least one of the three experiments.

**Figure 4:**
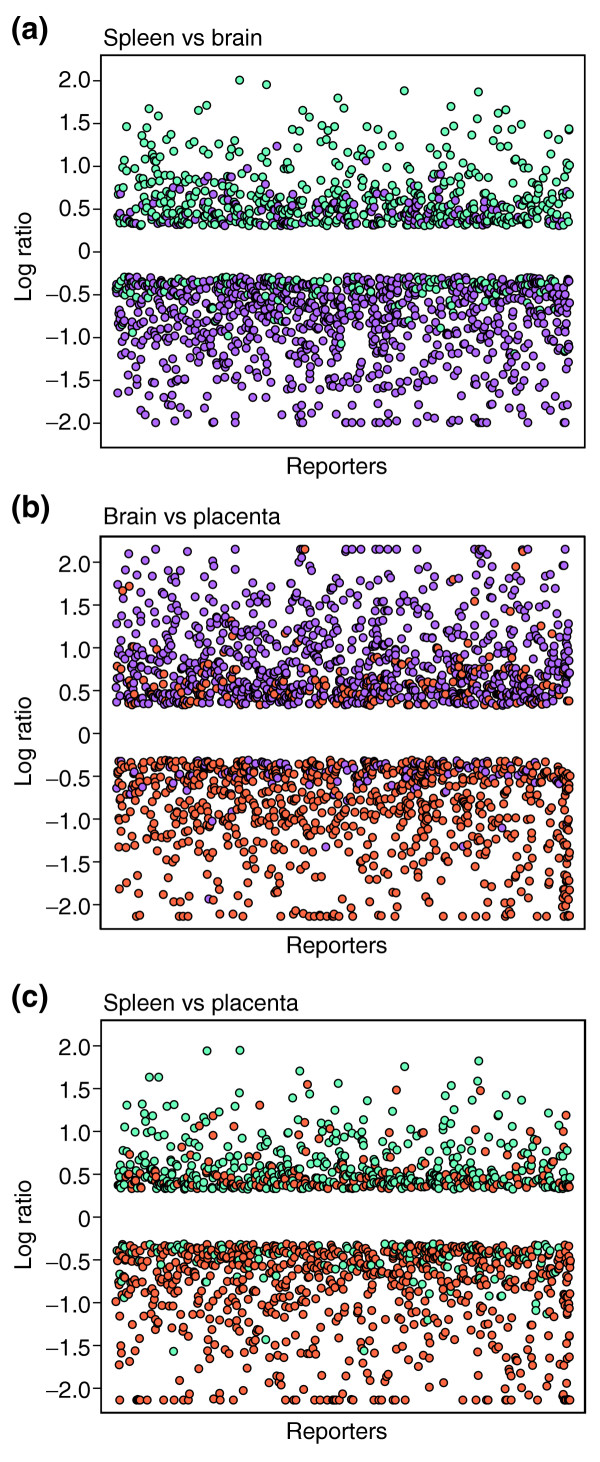
Tissue-specific gene-expression patterns measured using the rhesus macaque oligonucleotide microarray. To evaluate whether arrayed oligonucleotides were binding target sequences, microarrays were hybridized with probes derived from RNA isolated from spleen, brain, or placenta. Probes were paired in different combinations as indicated: **(a) **spleen vs brain; **(b) **brain vs placenta; **(c) **spleen vs placenta. Oligonucleotides that detected a difference in gene expression of twofold or more (*P *≤ 0.01) between two tissues are indicated as colored points and are displayed across the *x*-axis to facilitate visualization. The *y*-axis represents the log ratio for differentially expressed genes in each tissue comparison. Points corresponding to oligonucleotides derived from spleen ESTs are colored blue, those from brain are magenta, and those from placenta are orange. Thus, in the comparison depicted in **(a)**, genes more highly expressed in the spleen are indicated by points in the upper portion of the panel (and are predominantly sequences derived from the spleen cDNA library) and genes more highly expressed in the brain are indicated by points in the lower portion on the panel (and are predominantly sequences derived from the brain cDNA library). Plots were prepared using Spotfire.

Plots of the log-transformed ratios for genes in each experiment that showed an equal to or greater than twofold difference in expression between two tissues are shown in Figure 4. In each plot, points are colored according to the source library of the sequence used to derive the corresponding oligonucleotide. From this analysis, it is apparent that the majority of genes that were more highly expressed in the spleen correspond to sequences derived from the spleen cDNA library. Similarly, the majority of genes that were more highly expressed in the brain correspond to sequences derived from the brain cDNA library. These results show that a majority of the oligonucleotides were successfully binding target sequence. In addition, it is likely that many of the oligonucleotides that did not measure differential gene expression in these experiments are also successfully binding target sequences, as not all genes would be expected to be expressed in all tissues or to show differential levels of expression between the tissues analyzed.

## Discussion

Primate models are essential to the study of human biology and disease and to the development of new pharmaceutical products, many of which require primate testing before approval for use in humans. The closest living primate relatives to human are the chimpanzee and other great apes [33]. Human and chimp lineages diverged from a common ancestor 5-7 million years ago (Mya) and the genomes of the two species are highly conserved [4,24,34-36]. Experimental research using chimpanzees and other great apes is, however, significantly hampered by their size, maintenance costs, and endangered species status. The human-like qualities of the chimpanzee also make research using this animal generally unacceptable for ethical reasons. For the most part, chimpanzees are rarely used for invasive studies except, for example, when investigating diseases for which there is no other animal model (for example, hepatitis C infection) [37].

Old World monkeys, a group that includes macaque, baboon, and African green monkey, are our closest non-ape relatives. Old World monkeys and humans shared a common ancestor around 25 Mya, and the genomes of these organisms are highly conserved with human [33,35,38]. Furthermore, the biology of these organisms is such that they are an appropriate primate model for human physiology and disease. For this and other reasons, Old World monkeys are widely used in biomedical research, with members of the *Macaca *genus most frequently used [6].

We report here on the first phase of a study to sequence the rhesus macaque transcriptome. Our group has collected sequence data from 48,642 cDNA clones from nine animals and 11 tissues. For the current study, standard cDNA sequencing methods were used, with an emphasis on large clone-inserts and long sequence read lengths. Alternative methods could have been used for data collection that would have resulted in less 3'-end bias (for example, ORESTES [39]) or reduced redundancy in the collected data (for example, library normalization [40]).

We determined the average sequence divergence between human and macaque to be 2.21% for coding and 4.90% for noncoding sequence. An identical analysis of transcribed chimpanzee sequences demonstrated divergences of 1.70% and 2.35% for coding and noncoding sequence respectively. This is in comparison to a recently reported mean 1.44% divergence between human chromosome 21 and chimpanzee chromosome 22 over their entire length [4]. The continued analysis of sequence divergence between the macaque and human species will be important for translating data collected in this primate model to human biology. Recent evidence suggests that even minor inter-species sequence variation can result in large phenotypic differences between macaque models and human disease [8,41,42].

In addition, we have identified gene functional groups with higher than average sequence divergence at the amino-acid level. In one example, we observe 15% amino-acid sequence divergence between putative human and macaque orthologs of the cytidine deaminase APOBEC3C. Consistent with this observation, Sawyer *et al. *have reported evidence for accelerated evolution of the primate APOBEC gene family, probably under the selective pressure of viruses [20]. Members of this family (for example, APOBEC3G) have antiviral activity against lentiviruses and specifically against HIV [19]. APOBEC3G is packaged into nascent virions and delivered together with the viral genome into newly infected host cells. The cytidine deaminase cargo results in hypermutation of the replicating virus in target cells, thereby inhibiting virus infection. The Vif proteins of HIV and other lentiviruses bind APOBEC3G and inhibit its antiviral activity. However, the interaction between Vif and APOBEC3G is highly species and virus specific. HIV Vif can inhibit the function of human but not simian APOBEC3G [42]. Likewise, Yu and colleagues have recently reported that human APOBEC3B and APOBEC3C can inhibit SIV but not HIV-1 infection of human cells [43]. Our observation of poor sequence conservation between macaque and human APOBEC3C is consistent with a model of accelerated evolution under selective pressure for this gene family.

This dataset has further enabled us to conduct a preliminary analysis of nucleotide diversity within the *M. mulatta *species and the degree of divergence among *M. nemestrina*, *M. fascicularis*, and *M. mulatta*. Mean nucleotide divergence computed over 24 genes is 15.8 ± 12.5 × 10^-4^, approximately twofold greater than that computed for human transcribed sequences by several recent comprehensive studies [26,44]. Excess nucleotide diversity in macaque versus human is consistent with observations from other primate species. In general, numerous groups have observed increased nucleotide diversity in mitochondrial [45-47], sex chromosome [48-51], and autosomal DNA [28,52,53] sequences from chimpanzee, bonobo, and gorilla. Consistent with other primate species, this observation is likely to reflect a larger effective population size for macaque throughout evolution relative to human. Our analysis also confirms a high degree of sequence similarity among macaque species, with pairwise divergence estimates (0.380-0.588%) exceeding intraspecies heterozygosity. *M. mulatta *and *M. fascicularis *appear more closely related to each other than either one is to *M. nemestrina*, although these differences did not reach significance.

We describe a small number of macaque sequences without apparent human ortholog. Confirmation of this observation will require a complete sequence of the rhesus genome, but these preliminary data are consistent with recent comparative human-chimpanzee analyses demonstrating many small insertions/deletions and rearrangements between these species, some of which contain open reading frames or expressed sequences [4,29,54].

Finally, we report on the development of a first-generation rhesus-specific oligonucleotide microarray to support gene expression analyses of cells and tissues from this animal. Previously, investigators have used human DNA microarrays to measure gene expression changes in macaque tissues. Although the high degree of nucleotide sequence identity between humans and macaques makes this cross-species hybridization feasible, it is not clear to what extent sequence divergence between these species may affect gene expression measurements. Our observation of a small number of macaque sequences without apparent human ortholog also suggests the importance of using species-specific arrays. The rhesus microarray should therefore facilitate the use of the macaque model for future gene expression profiling experiments and may also be useful for studying similarities and differences in gene expression between macaque and human tissues [55]. To this end, we have included on the microarray 1,014 human oligonucleotide sequences, many of which were chosen because they are orthologs of macaque sequences also present on the array. In addition, because we anticipate this array will be widely used for infectious disease research, many of the human sequences have relevance to cytokine signaling, apoptosis, or the immune response, and we have included oligonucleotides corresponding to genes from 20 different viruses.

## Conclusion

While the macaque species are widely used primate models of human physiology and disease, there are few species-specific genomic resources available to the research community. Furthermore, the applicability of the macaque model to human disease will be highly dependent on the degree of sequence divergence between macaque and human, among the macaque species, and among animals of divergent geographic origin. Comprehensive genome-wide analysis has begun to characterize inter-species differences and to provide resources, such as the rhesus-specific microarray, that will enable a more efficient use of this model organism in the future.

## Materials and methods

### Animal tissues

Animal tissues and blood were provided by the Tissue Distribution Programs of the Washington and Oregon National Primate Research Centers. All *M. mulatta *animals were of Indian origin and had wild-caught parents. No mitochondrial DNA or major histocompatibility complex (MHC) typing was performed.

### RNA isolation

Rhesus macaque tissues used for RNA isolation were harvested at necropsy and immediately placed in RNAlater stabilization and storage solution (Ambion). Tissues were then homogenized in Solution D [56] either by hand or mechanically using a Polytron tissue homogenizer, and total RNA was isolated by guanidinium isothiocyanate-phenol-chloroform extraction and further purified using RNeasy purification columns (Qiagen). Extraction of mRNA was performed using the FastTrack 2.0 mRNA extraction kit (Invitrogen). RNA quality and quantity were determined by spectrophotometry and by capillary electrophoresis using an Agilent Technologies BioAnalyzer.

### cDNA library construction and sequencing

cDNA libraries were constructed using two alternative methods. Spleen mononuclear lymphocyte, brain, lung, activated PBMC, and two placental libraries (from male and female fetuses) were constructed with the Uni-ZAP cDNA library construction kit (Stratagene) using 3-5 μg high-quality mRNA for each library. Clones were isolated by ampicillin resistance and grown in 96-well plates containing LB-ampicillin medium. Liver, duodenum, ileum, jejunum, testes, ovary, and activated PBMC libraries were constructed with the CloneMiner cDNA construction kit (Invitrogen), again using 3-5 μg high-quality mRNA for each library. Clones were isolated by kanamycin resistance and grown in 96-well plates containing LB-kanamycin medium. All libraries were constructed with size-fractionated RNA, resulting in a mean insert size of approximately 1.5 kbp for each library as determined by PCR. Clone inserts were sequenced from the vector-insert junction distal to the poly(A) tail such that most resulting sequences do not include a poly(A) tail at their 3' terminus. For each clone, inserts were amplified by PCR directly from 0.2 μl of the glycerol stock using the following primers: for the Stratagene pBluescript SK (+/-) vector: 5' -CCCTCACTAAAGGGAACAAAA (the sequencing primer) and 5' -CACTATAGGGCGAATTGGGTA; for the Invitrogen pDONR222 vector: 5' -GACGTTGTAAAACGACGGC (the sequencing primer) and 5' -GCCAGGAAACAGCTATGACC. PCR products were sequenced using standard fluorescent dye-terminator chemistries on an Applied Biosystems 3700 capillary sequencer.

### Sequence data analysis

cDNA sequences were first base-called using a modified version of the phred algorithm ([9,57] and C.M., unpublished data) and then screened for cloning vector, lambda-phage, and *E. coli *contamination using the program cross_match [9]. Sequences exhibiting multiple cloning sites or any contamination with lambda-phage or *E. coli *sequence were removed from further analysis. Leading- and trailing-cloning vector sequence was masked from all remaining sequences. Putative polyadenylation was identified by the presence of a consensus mammalian polyadenylation signal [10] followed by an (A)_10 _tract within 50 bp. The remaining sequences were analyzed using MEGABLAST [13] against rhesus mitochondrial sequence (GenBank accession AY612638.1) and against the human mRNA RefSeq collection [12] to identify putative human orthologs. Sequences with a significant match to any putative human ortholog were selected for GenBank submission. Of these sequences, 36,921 met minimum quality criteria for submission to GenBank. Each sequence was assigned a putative human ortholog if there was a unique maximally-scoring match by MEGABLAST bit score comparison; sequences with multiple maximal matches were not assigned an ortholog. Sequences with no significant RefSeq match were further analyzed by similar MEGABLAST comparisons against EST, genomic, and protein databases. Sequences were also analyzed for human repetitive sequence families using cross_match.

### Rhesus-human similarity analyses

For the nucleotide comparisons, macaque sequences were selected for inclusion that were assigned a putative human ortholog and that spanned the human ortholog's final coding nucleotide by at least 150 nucleotides in each direction (as determined by initial MEGABLAST results). Selected sequences were then realigned independently against two subsequences of the corresponding human ortholog: one containing the final 150 coding nucleotides and the other containing the first 150 nucleotides in the 3' UTR. Results across all sequences were grouped by ortholog and the maximal bit score match in each region selected. For the amino-acid comparisons, macaque sequences were selected that had been assigned a putative human ortholog and contained at least 450 high-quality bases spanning the 3' end of the putative coding region (as determined by initial MEGABLAST results). Selected sequences were realigned independently (by translated MEGABLAST) against the protein sequence corresponding to the assigned ortholog. Results from all sequences were grouped by ortholog and by the maximal bit-score match selected. Grouping by protein classes was completed by cross-reference of each orthology against its GO biological process assignment.

### Genomic PCR analysis of macaque sequences

PCR reactions (10 μl) included 0.132 U of Platinum Taq polymerase (PerkinElmer), 0.5 μM each primer, 0.132 μM each dNTP, 13 mM Tris-HCl (pH 8.4), 33 mM KCl, and 1 mM MgCl_2_. Thermal cycling was conducted in a PerkinElmer 9700 as follows: 95°C for 5 min (one cycle); 95°C for 30 sec, 55°C for 30 sec, 72°C for 1 min (40 cycles); and 72°C for 1 min (one cycle). Amplifications were evaluated under a variety of annealing temperatures between 55-60°C. Primer sequences are as follows (target/forward/reverse):

CX078591: 5'-GGAGAATCCAGTTAACGGCT-3', 5'-CTCTCATCCAGCCTAACGTG-3';

CX078602: 5'-GTTTTCAAAGAGCCCAGCAA-3', 5'-CTTTGGCATAGCTTCGGTTC-3';

CX078598: 5'-GGCAACAAGTGGGAATCAAC-3', 5'-GAGGAATCGGGATGGTCATA-3';

CB552301: 5'-CCTCCTTGGACTTGGACCTT-3', 5'-AGGACAGGAGTCTTGCCAAA-3';

CB555845: 5'-GTCAACAGGCTGGCATTTTA-3', 5'-CAATTATTGACCCCAAGGCTA-3';

CX078592: 5'-CAAAGCCATCAGACAGCAGA-3', 5'-GAGACCAGGAAAGTCGAAGG-3';

CB552531: 5'-CTGGAATAAGGCCAGAAGCA-3', 5'-ATTCCTCAGGTCTGGTGGAG-3';

CX078596: 5'-CCTCATGGTGTGGCTATGTG-3', 5'-ACACAAGGCGAGCTCTGGTA-3';

OAS1: 5'-GAGCCAAGAAGTACAGATGC-3', 5'-AGGACAGAGCTGTCCAATAG-3'.

### Oligonucleotide microarray design

To design sequences for a rhesus macaque oligonucleotide microarray, we began with over 20,000 EST reads from clones derived from six cDNA libraries (spleen mononuclear lymphocyte, brain, lung, PBMC, and male and female placenta). After base-calling and quality filtering, sequences were processed using TIGR clustering tools [32] and compared by BLASTN with Human UniGene cluster representatives (build 167). High-quality reads that had at least one strong hit to Human UniGene were carried forward for oligonucleotide design. An additional 584 rhesus macaque sequences were provided by Robert Norgren (University of Nebraska) and Eliot Spindel (Oregon National Primate Research Center).

Oligonucleotides based on these sequences were designed by Agilent Technologies. Repeat sequences were identified, masked, and excluded. Candidate oligonucleotides were selected from the 3' end of each target sequence, filtered according to optimal base-composition profiles, and screened on the basis of predicted hybridization properties and potential cross-hybridization with other sequences. Four 60-mer oligonucleotides were initially designed for each target sequence that passed quality-control checks. To estimate specificity, each oligonucleotide was compared with Human UniGene build 167. Oligonucleotides with strong similarity to more than one UniGene cluster were then manually checked for cross-hybridization against the July 2003 assembly of the human genome (hg16) using the University of California Santa Cruz Genome Browser [58]. Oligonucleotides that hit more than one region of the human genome were discarded as ambiguous.

Because target sequences were not filtered by annotation before oligonucleotide design, multiple oligonucleotides were often designed to different regions of the same gene. Oligonucleotides were therefore mapped to UniGene cluster sequences and two high-scoring oligonucleotides were selected for each underlying transcript represented. This resulted in a final set of 7,973 macaque oligonucleotides representing approximately 3,944 unique genes. In addition to the macaque oligonucleotides, 1,014 oligonucleotides corresponding to 894 human genes and 96 oligonucleotides corresponding to genes from 20 different viruses were also selected for inclusion on the microarray. Duplicate 60-mer oligonucleotides were arrayed onto glass slides by Agilent Technologies. The array is commercially available from Agilent Technologies, Agilent Microarray Design Identification (AMADID) Number 012650 [59].

### Labeled probe synthesis and microarray hybridization

For microarray analysis, total RNA was extracted from spleen, brain, and placental tissues. Each tissue was obtained from a different animal. RNA quality and quantity were determined by spectrophotometry and by capillary electrophoresis using an Agilent Technologies BioAnalyzer. Labeled cRNA probes were generated using the Low Input RNA Probe Synthesis Kit (Agilent Technologies) according to the manufacturer's protocol for 11K postage-stamp oligonucleotide microarrays. The probes were hybridized in replicate to the rhesus macaque oligonucleotide microarray according to the manufacturer's protocol. Slides were scanned with an Agilent DNA microarray scannerand image analysis was performed using Agilent feature extraction software. All data were entered into a custom-designed gene-expression database, Expression Array Manager, and then uploaded into Resolver 4.0 (Rosetta Biosoftware) and DecisionSite for Functional Genomics (Spotfire) for analysis.

### Data submission and databases

There are 36,921 GenBank accession numbers associated with this manuscript. They are cross-referenced and publicly available at the project website [11]. Expression microarray data have been submitted to EBI ArrayExpress, accession number E-TABM-9.

## Additional data files

The following additional data are available with the online version of this paper. Additional data file 1 is a table of chimpanzee versus human sequence identity. Additional data file 2 is a figure showing the distribution of coding and noncoding sequence similarity between chimpanzee and human.

## Supplementary Material

Additional File 1 Chimpanzee versus human sequence identity. Chimpanzee cDNA and EST sequences in GenBank were compared to human RefSeqs to provide a dataset comparable to the macaque-human results described herein. Due to the paucity of expressed chimpanzee sequence data in GenBank, the analysis could only be performed for 134 human-chimp pairs. Data for sequence identity between chimpanzee and human for these genes are noted in the table. Macaque-human identities are also reported for the same genes where available. Click here for file

Additional File 2 Distribution of coding and noncoding sequence similarity between chimpanzee and human. A histogram showing the degree of nucleotide sequence similarity between chimpanzee and human for coding (blue) and noncoding (3' UTR, yellow) transcribed sequence. EST and cDNA sequences (N = 134, Additional data file 1) were selected that cross a well defined stop codon and that provide concurrent sampling of 150bp of sequence both proximal and distal to the stop. The best human match for each chimpanzee sequence was identified and compared using MegaBlast. Click here for file
